# Effectiveness of Oral Roflumilast Among Chronic Obstructive Pulmonary Disease Patients to Prevent Exacerbations

**DOI:** 10.7759/cureus.110036

**Published:** 2026-06-01

**Authors:** Talha Laique, Hina Latif, Maimoona Z Khan, Zainab Taseer, Asia Firdous, Hira B Jabbar, Usama Sajid

**Affiliations:** 1 Internal Medicine, Mayo Hospital, Lahore, PAK; 2 Internal Medicine, Mayo Hospital, King Edward Medical University, Lahore, PAK; 3 Pharmacology, Lahore Medical and Dental College, Lahore, PAK; 4 Internal Medicine, Shaikh Khalifa Bin Zayed Al-Nahyan Medical and Dental College, Lahore, PAK

**Keywords:** copd, efficacy, number of exacerbations, phosphodiesterase 4 (pde4) inhibitor, pulmonary function tests (pfts)

## Abstract

Background

Chronic obstructive pulmonary disease (COPD) is a common and severely incapacitating respiratory illness that is characterized by ongoing, non-reversible airflow restrictions. Various medical treatment options include bronchodilators, anti-cholinergic drugs, and steroids. This study aimed to evaluate the effectiveness of oral roflumilast in addition to standard therapy among COPD patients to prevent exacerbations and improve pulmonary function tests (forced expiratory volume in one second (FEV1) and forced vital capacity (FVC)).

Methodology

This quasi-experimental study enrolled 80 moderate-to-severe COPD patients through non-probability sampling. They were divided into two groups. In Group A, participants received standard treatment as per the operational definition for six months. In Group B, participants received once-daily roflumilast, 500 µg, while maintaining background medications of COPD as per the operational definition for six months. Quantitative variables, including age, post-bronchodilator FEV1, and FVC, were expressed as mean ± SD. An independent-sample t-test was used to compare the outcome variables (FEV1 and FVC) between the two groups. The number of COPD exacerbations was analyzed using an appropriate statistical test, such as the Mann-Whitney U test. All the data were analyzed using SPSS version 28.0.

Results

Both groups showed a significant increment in mean FEV1 and FVC from day zero to six months; however, mean FEV1 and FVC were significantly higher for Group B patients from the fourth to sixth months. A decreasing trend was observed in the frequency of exacerbations in both groups. At the fifth and sixth months of treatment, Group B patients showed significantly lower frequency of exacerbations compared to Group A patients.

Conclusions

Oral roflumilast as an adjunct to standard COPD therapy improved clinical outcomes by enhancing pulmonary function (particularly FEV1) and reducing exacerbation frequency. Reduction in recurrent exacerbations may contribute to better symptom control, improved quality of life, and decreased healthcare burden. Therefore, roflumilast 500 µg once daily may be considered a useful add-on option in selected COPD patients, although larger studies with longer follow-up are warranted to establish long-term benefits.

## Introduction

Chronic obstructive pulmonary (COPD) disease is a common and severely incapacitating respiratory illness that is characterized by ongoing, non-reversible airflow restrictions [1]. It includes a range of respiratory conditions, such as emphysema and chronic bronchitis, which are usually accompanied by progressive airflow obstruction and respiratory symptoms such as coughing, sputum production, and dyspnea [2]. Globally, COPD is the primary cause of illness and mortality, placing a high burden on both individuals and healthcare systems. According to a previous estimate, this obstructive disorder is the fourth leading cause of death globally. However, according to the World Health Organization (WHO) prediction, it will be the third leading cause of death by 2030 [3]. According to previous studies, COPD and asthma accounted for almost 8% of all disability-adjusted life years. COPD prevalence in Pakistan has been documented to be 2.1%, whereas the prevalence of asthma has been reported to be 4.3% [2,3].

It has been estimated that in low-middle-income countries (LMICs) such as Pakistan, India, Sri-Lanka, and Afghanistan, among others, common pulmonary disorders such as COPD and asthma remain underdiagnosed due to a lack of medical facilities [4]. According to previous studies, several therapeutic agents belonging to different classes play a vital role in treating conditions such as COPD [4,5]. In case of infective exacerbations, antibiotics such as azithromycin or penicillin can be used. However, as there is an increase in resistant cases of COPD towards standard treatment, new treatment options have been explored. Due to medical advancements, new therapeutic agents have been introduced in the market following Food and Drug Administration (FDA) approval for the treatment of common as well as rare diseases. A new class of drug, a phosphodiesterase-4 (PDE-4) inhibitor (roflumilast), has been approved for the treatment of severe COPD following exacerbations. It increases intracellular cAMP levels and thereby reduces airway inflammation in COPD [6]. A previous study revealed that acute exacerbations of COPD result from a combination of bacterial and viral infections in around 70% of cases, thus contributing to its high mortality among patients [7].

Pakistan is overburdened by COPD due to a lack of medical facilities and research among the local population regarding the effective role of PDE-4 inhibitors in the management of COPD. As roflumilast has a good safety profile, we planned to evaluate the effectiveness of oral roflumilast in addition to standard therapy among COPD patients to prevent exacerbations and improve pulmonary function tests (forced expiratory volume in one second (FEV1) and forced vital capacity (FVC)).

## Materials and methods

After obtaining approval from the Institutional Review Board of King Edward Medical University (approval number: 1040/RC/KEMU dated 17/12/22) and the Advanced Studies & Research Board (approval number: 372 /KEMU/23 dated 11/01/23), a total of 80 cases fulfilling the inclusion and exclusion criteria were enrolled. This was a quasi-experimental, open-label study conducted using non-probability sampling from the Department of Medicine and Chest Medicine, Mayo Hospital, Lahore. This study was conducted from April 2023 to October 2023. After obtaining informed written consent, demographic data (including name, age, gender, occupation, smoking status) were noted on a specially designed proforma. Moderate (with frequent exacerbations ≥3), severe, and very severe COPD patients aged 40-80 years of either gender were enrolled through non-probability sampling. Patients with pregnancy, lactation, interstitial lung disease, human immunodeficiency virus/immunocompromised states, pulmonary hypertension due to other causes, decompensated chronic liver disease, end-stage renal disease, or congestive cardiac failure were excluded based on medical records. A total sample size of 80 patients was estimated using 5% level of significance, 90% test power, with an expected mean value of FVC. Group A (only standard treatment): 45.26 ± 14.88, and group B (Rofluminlast plus standard treatment): 51.26 ± 11.05 [8].

All patients were divided into the following two groups: Group A (control group) and Group B (treatment group). In Group A, participants received standard treatment as per the operational definition for six months [8]. In Group B, participants received once-daily roflumilast, 500 µg, while maintaining background medications (bronchodilators, anti-cholinergic drugs, and steroids) of COPD as per the operational definition for six months [8]. Baseline clinical parameters of all patients were noted. All variables (pulmonary function tests (FEV1 and FVC) and number of exacerbations) were measured at the start of the study and were repeated at monthly intervals for six months. All data were collected by the researchers.

All data were analyzed using SPSS version 28.0 (IBM Corp., Armonk, NY, USA). Quantitative variables, including age, post-bronchodilator FEV1, and FVC, were expressed as mean ± standard deviation (SD), while qualitative variables, including gender, smoking status, and stages of COPD, were presented as frequencies and percentages (%). An independent-samples t-test was used to compare continuous outcome variables (FEV1 and FVC) between the two groups. The number of COPD exacerbations, being count data, was analyzed using an appropriate statistical test, such as the Mann-Whitney U test. A p-value <0.05 was considered statistically significant.

## Results

Demographic parameters, including age, gender distribution, and smoking status, are shown in Table 1. The mean age of the patients was 63.80 ± 8.74 and 67.80 ± 9.42 years in Group A and Group B, respectively.

**Table 1:** Demographic parameters among enrolled patients (n = 80). N = frequency; % = percentage; SD = standard deviation

Parameters	Group A	Group B
Age (years)		
Mean ± SD	63.80 ± 8.74	67.80 ± 9.42
Minimum	52	52
Maximum	75	80
Gender distribution, N (%)		
Male	18 (45%)	25 (62.5%)
Female	22 (55%)	15 (37.5%)
Smoking status of patients, N (%)		
Yes	24 (60%)	26 (65%)
No	16 (40%)	14 (35%)

Most patients in both groups had stage 3 COPD, followed by stage 4 and stage 2 disease, as shown in Table 2.

**Table 2:** Stage of COPD. The chi-square test was applied. N = frequency; % = percentage; SD = standard deviation; COPD = chronic obstructive pulmonary disease

Parameters	Group A	Group B	Total
Stages	N (%)	N (%)	N (%)
Stage 2	10 (25%)	6 (15%)	16
Stage 3	22 (55%)	21 (52.5%)	43
Stage 4	8 (20%)	13 (32.5%)	21
Total	40	40	80
X^² ^value	2.214		
P-value	0.331		

From the fourth to sixth month, mean FEV1 was significantly higher for Group B patients. Both groups showed a significant increment in mean FEV1 from day zero to six months. However, the mean increment in FEV1 was significantly higher for Group B patients (Table 3).

**Table 3:** FEV1 in study groups. *: Statistically significant (p-value ≤0.05); independent t-test was applied. FEV1 = forced expiratory volume in one second

FEV1 (L)	Group A	Group B	t-value	P-value
Day 0	1.13 ± 0.36	1.07 ± 0.15	0.97	0.334
1^st^ month	1.16 ± 0.34	1.10 ± 0.16	0.85	0.397
2^nd^ month	1.17 ± 0.36	1.16 ± 0.17	1.19	0.236
3^rd^ month	1.19 ± 0.31	1.28 ± 0.21	-1.42	0.159
4^th^ month	1.24 ± 0.34	1.38 ± 0.23	-2.15	0.036*
5^th^ month	1.35 ± 0.30	1.51 ± 0.22	-2.68	0.009*
6^th^ month	1.47 ± 0.29	1.61 ± 0.23	-2.28	0.026*
P-value	<0.001*	<0.001*		

Table 4 describes the mean FVC in both treatment groups from day zero till the sixth-month follow-up. From the fourth month until the sixth month, the mean FVC was significantly higher for Group B patients. Both groups showed a significant increment in mean FVC from day zero to the sixth month. However, the mean increment in FVC was significantly higher for Group B patients.

**Table 4:** FVC in study groups. *: Statistically significant (p-value ≤0.05); independent t-test was applied. FVC = forced vital capacity

FVC (L)	Group A	Group B	t-value	P-value
Day 0	2.35 ± 0.64	2.46 ± 0.60	-0.82	0.415
1^st^ month	2.37 ± 0.62	2.51 ± 0.59	-1.04	0.300
2^nd^ month	2.45 ± 0.63	2.59 ± 0.60	-1.00	0.318
3^rd^ month	2.53 ± 0.61	2.68 ± 0.60	-1.17	0.246
4^th^ month	2.49 ± 0.58	2.81 ± 0.64	-2.32	0.023*
5^th^ month	2.62 ± 0.60	2.89 ± 0.64	-1.99	0.050*
6^th^ month	2.69 ± 0.58	2.97 ± 0.64	-2.08	0.040*
P-value	<0.001*	<0.001*		

A progressive reduction in exacerbation frequency was observed in both groups during the sixth-month follow-up. However, Group B showed significantly fewer exacerbations compared to Group A at the first, second, third, fifth (p < 0.001), and sixth months (p = 0.018), as shown in Table 5.

**Table 5:** Number of exacerbations in treatment groups. *: Statistically significant (p-value ≤0.05); Mann–Whitney U test was applied.

	Day 0		1^st ^month		2^nd ^month		3^rd ^month		4^th^ month		5^th^ month		6^th^ month	
	A	B	A	B	A	B	A	B	A	B	A	B	A	B
2 episodes	-	-	-	-	-	-	-	-	-	-	25%	32.5%	25%	47.5%
3 episodes	-	-	-	-	-	-	45%	32.5%	45%	50%	40%	67.5%	65%	52.5%
4 episodes	-	-	25%	57.5%	25%	50%	45%	67.5%	45%	50%	35%	-	10%	-
5 episodes	90%	85%	65%	42.5%	65%	50%	10%	0%	10%	-	-	-	-	-
6 episodes	10%	15%	10%	0%	10%	0%	-	-	-	-	-	-	-	-
P-value	0.499		0.003*		0.016*		0.037*		0.163		<0.001*		0.018*	
Mann-Whitney U test	748		432.5		491		536.5		671		298		482.5	

## Discussion

For Pakistani individuals with COPD of any severity, this quasi-experimental study is the first to fully assess adding a PDE-4 inhibitor (roflumilast) to conventional medication. Roflumilast selectively inhibits PDE-4, increasing intracellular cAMP levels and thereby reducing airway inflammation in COPD. Patients with moderate-to-very severe COPD were the main focus because they are the group with the highest clinical need. These patients require combination therapy to control their disease. Targeted anti-inflammatory therapies were effective for this group of patients, according to recent research using a variety of bronchodilators. As roflumilast has anti-inflammatory properties, it was hypothesized that if oral roflumilast was added to the standard treatment of COPD, there might be better improvement in respiratory rate, FEV1 by spirometry, and stage of COPD. Pakistan ranks fourth among high-burden countries for COPD and sixth among countries with the highest number of refractory COPD due to resistance to bronchodilators/steroids, according to a WHO COPD report [1]. Refractory COPD treatment has multiple reasons, such as poverty, poor literacy rate, and less awareness about the disease and its consequences. Given the increasing multidrug-resistant COPD cases, the use of roflumilast (500 µg) daily was added to the standard treatment protocol of seriously ill COPD patients.

The age (mean ± SD) of the enrolled COPD patients in our study was 63.80 ± 8.74 years in Group A and 67.80 ± 9.42 years in Group B (Table 1). The age of patients ranged between 52 and 80 years. In a previous study, the mean age of enrolled COPD patients in Group A (treatment group) was 64.3 years, while 61.2 years in the standard group (Group B) [9]. Thus, the mean ages (years) were consistent with the above-mentioned study. Another study showed that the mean age among the COPD patients was 63 years in the placebo group, while 65 years was noted in the roflumilast group [10]. Thus, age groups in the present study were in line with previously reported studies.

Both males and females were recruited in this trial, as in other studies. In the present study, Group A had 18 (45%) males and 22 (55%) females, while in Group B, 25 (62.5%) were male patients and 25 (37.5%) were female patients (Table 2). Our results showed almost equal prevalence of COPD among males and females, while according to many previous studies, males were more affected by COPD globally as well as in Pakistan [11,12].

Table 3 describes the mean FEV1 in both treatment groups from day zero to the sixth-month follow-up. From the fourth month onward till the sixth month, the mean FEV1 was significantly higher for Group B patients. Both groups showed a significant increment in mean FEV1 from day zero to the sixth month. However, the mean increment in FEV1 was significantly higher for Group B patients. Similarly, a previous study assessed the comparative effectiveness of roflumilast in reducing exacerbations and improving FEV1 in patients with severe COPD and chronic bronchitis. Their results showed significant improvement in FEV1 (mL), with an average increase of 40 mL compared to placebo [13]. Another study evaluated the efficacy of roflumilast in improving lung function (FEV1) and reducing exacerbations over 12 months. Their results showed improvement in FEV1 by an average of 50 mL in the roflumilast group compared to placebo [14]. Hence, the findings of our study in terms of FEV1 improvement were in line with the above-mentioned studies.

Table 4 describes the mean FVC in both treatment groups from day zero to the sixth-month follow-up. From the fourth month onward to the sixth month, mean FVC was significantly higher for Group B patients. Both groups showed a significant increment in mean FVC from day zero to the sixth month. However, the mean increment in FVC was significantly higher for Group B patients. Similar results were seen in a previous study that showed improvement in FVC among patients treated with roflumilast for four months. In their study, FVC before treatment was 46.54 + 11.71, while after four months of treatment with roflumilast, it was 51.26 ± 11.05 (p = 0.04) [9]. Another study from 2018 showed improvement in symptoms and FVC among patients treated with roflumilast for six months [15]. Hence, our results were in line with the above-mentioned studies.

Table 5 describes the number of exacerbations in both treatment groups from day zero until the sixth-month follow-up. At the first, second, and third months, the number of exacerbations showed a significant difference between groups. The frequency of exacerbations was higher in Group A patients compared to Group B patients. A decreasing trend was seen in the frequency of exacerbations in both treatment groups. At the fifth and sixth months, a significant difference was seen in the frequency of exacerbations between groups (fifth month: p-value<0.001, sixth month: p-value = 0.018). Group B patients had a significantly lower frequency of exacerbations compared to Group A patients. Several similar clinical trials have demonstrated that roflumilast effectively reduces the frequency of exacerbations in patients with severe COPD. Another study evaluated roflumilast in combination with a long-acting beta-agonist versus placebo. Their results showed that roflumilast significantly reduced the rate of moderate-to-severe exacerbations [10,12]. A 2020 study assessed the impact of roflumilast on patients with a history of frequent exacerbations. The study findings indicated a substantial reduction in exacerbation rates in the roflumilast group compared to placebo, demonstrating its effectiveness in this high-risk population [16]. Thus, the results of our study were in line with the above-mentioned studies that indicated a declining trend in the frequency of exacerbation of COPD. However, a few patients who received roflumilast suffered weight loss and insomnia, as documented previously.

Strengths

The results of the present study highlighted the effectiveness of roflumilast in the treatment of COPD among the local population in addition to standard treatment. It helped improve the management and clinical outcomes of COPD patients. A progressive reduction in exacerbation frequency was observed in both groups during the sixth-month follow-up.

Limitations

This study has several limitations, including a small sample size, a single trial center, and financial constraints due to a lack of resources. For logistical reasons, we limited follow-up to once a month for sis months. Other limitations included its quasi-experimental, non-randomized, open-label design, which may have introduced selection and assessment bias. Potential confounding factors such as concomitant COPD medications, hospitalization status, and regression to the mean were not controlled, thus limiting the generalizability of the findings. However, there was no missing data and loss to follow-up.

## Conclusions

The use of oral roflumilast as an adjunct to standard therapy was effective in improving clinical outcomes among patients with COPD. Patients receiving roflumilast demonstrated improvement in pulmonary function, particularly in FEV1, along with a reduction in the frequency of COPD exacerbations. As recurrent exacerbations contribute substantially to disease progression, worsening quality of life, repeated hospital admissions, and increased healthcare burden, reducing their occurrence remains an important therapeutic goal in COPD management. These findings suggest that oral roflumilast provided additional benefit when combined with conventional COPD treatment by targeting inflammatory pathways involved in disease progression. Improvement in lung function and reduction in exacerbation frequency contributed to better symptom control and overall clinical outcomes in COPD patients. Based on these findings, it is recommended that roflumilast 500 µg once daily may be considered as an add-on therapy in routine clinical practice for appropriately selected COPD patients to enhance disease control and improve patient outcomes. Further studies with larger sample sizes and longer follow-up durations may provide additional insight into its long-term effectiveness and broader clinical benefits.
